# Complete genome sequence of *Bacillus safensis* strain BS22LVI isolated from the intestine of whiteleg shrimp, *Litopenaeus vannamei*

**DOI:** 10.1128/mra.00119-25

**Published:** 2025-05-27

**Authors:** Ju-Yeop Lee, Yoonhang Lee, Jiyeon Park, Hyo-Young Kang, Young-Ung Heo, Jung Min Lee, Thanh Luan Nguyen, Do-Hyung Kim

**Affiliations:** 1 Department of Aquatic Life Medicine, College of Fisheries Sciences, Pukyong National University 34998, Busan, South Korea; 2 Department of Science, Technology and International Affairs, HUTECH University 638418, Ho Chi Minh City, Vietnam; Rochester Institute of Technology, Rochester, New York, USA

**Keywords:** *Bacillus safensis*, AHPND, shrimp, probiotics, antimicrobial activity, quorum quenching, *Bacillus*, bacteria

## Abstract

We report the complete genome sequence of *Bacillus safensis* strain BS22LVI, isolated from the mid-hindgut of whiteleg shrimp (*Litopenaeus vannamei*) in Korea. The 3,824,412 bp circular genome has a G + C content of 41.65% and encodes 3,828 predicted coding sequences, including genes associated with antimicrobial activity and quorum quenching.

## ANNOUNCEMENT

Acute hepatopancreatic necrosis disease (AHPND), caused by *Vibrio* species harboring the *pirAB*-encoding pVA1-type plasmid, has severely impacted shrimp aquaculture worldwide (1). Due to the limited efficacy of conventional antibiotics (2), *Bacillus* spp. have been explored as promising probiotic candidates to combat AHPND (3). In this study, *Bacillus safensis* strain BS22LVI was isolated from the mid-hindgut of healthy live whiteleg shrimp (~25 g) obtained from a farm in Geoje Island, South Korea. The gut contents were homogenized in sterile phosphate-buffered saline, serially diluted, and spread on De Man, Rogosa, and Sharpe agar (Oxoid, USA). After incubation at 28°C for 24 h, morphologically distinct colonies were selected and purified.

Genomic DNA was extracted using the DNeasy Blood & Tissue Kit (Qiagen, Germany). 16S rRNA (4) and *gyrB* genes (Table 1) were PCR amplified using specific primers. BLASTn analysis revealed 97.2% and 98.3% identity with *B. safensis* FO-36B (CP010405.1), respectively. Antibacterial activity against *Vibrio parahaemolyticus* and *Vibrio harveyi* was confirmed by cross-streaking (5). In addition, quorum-quenching activity was demonstrated using a standard agar well-diffusion assay against N-acylhomoserine lactones (6). The isolate was stored at −80°C in brain-heart infusion broth (BHIB; Oxoid, USA) with 10% glycerol.

**TABLE 1:** Primers used for identification

Primer/probe	Primer sequence (5′→3′)		Target gene	References
*B. safensis*-F	ACGTTGTAACAATGTCATCTTTGA	95°C for 30 seconds (initial denaturation), followed by 35 cycles of 94°C for 3 seconds, 58°C for 30 seconds, and 72°C for 1 min, 72°C for 10 min (final extension)	*gyrB*	This study
*B. safensis*-R	AAACGCGGCAACTTTGTCAG			

For genome sequencing, a single colony was streaked on BHIA, incubated at 28°C for 24 h, and then inoculated into 5 mL of BHIB. The culture was grown at 28°C with shaking at 200 rpm for 24 h. Genomic DNA was extracted using the DNeasy Blood & Tissue Kit (Qiagen, Germany) following the manufacturer’s instructions. High-molecular-weight DNA was quantified using a Qubit 3.0 fluorometer (Invitrogen, USA) with the Qubit dsDNA HS Assay Kit.

The sequencing library was prepared with the ligation sequencing kit SQK-LSK109 and barcoding kit (Oxford Nanopore Technologies [ONT], UK). High-molecular-weight DNA was used without shearing or size selection. Sequencing was carried out on a MinION device using an R10.4.1 flow cell (ONT), and basecalling was performed with Guppy version 6.5.7 (high-accuracy model) (7). Reads were trimmed using Porechop version 0.2.4 (8) and assembled *de novo* using Unicycler version 0.5.0 (9). Assembly quality was assessed using QUAST version 5.2.0 (10), and genome annotation was performed with the NCBI Prokaryotic Genome Annotation Pipeline version 1.14.6 (11). Default parameters were used for all software unless otherwise specified.

The complete genome of BS22LVI consists of a single circular chromosome of 3,824,412 bp, with a G + C content of 41.65%. The assembly yielded one contig with an *N*_50_ of 3,808,502 bp and 35× coverage. A total of 3,828 coding sequences and 110 RNA genes were predicted. The strain exhibited 97.35% average nucleotide identity with *B. safensis* ATCC BAA-1126, a type strain.

Several biosynthetic gene clusters were identified, including genes encoding bacteriocins such as *skfA* (subtilosin) (12)*, uviB* (bacilysin) (13)*, ltnA* (lacticin3147) (14)*, eriS* (ericin) (15), and *bacA* (bacteriocin T8) (16). In addition, *ytnP,* encoding a lactonase responsible for degrading quorum-sensing signals, was identified, supporting the genetic basis for quorum-quenching activity (17). In conclusion, the genome sequence of BS22LVI provides insights into antimicrobial and quorum-quenching capabilities. Although antibacterial activity was confirmed against *V. parahaemolyticus* and *V. harveyi*, further studies are needed to evaluate its broader probiotic potential in shrimp aquaculture.

## Data Availability

Raw sequences of reads were submitted to NCBI SRA with accession numbers SRX27674927 under BioProject number PRJNA1204170 and BioSample number SAMN46014827. The assembled genome sequences of the *Bacillus safensis* strain BS22LVI were deposited in GenBank with accession number CP180263.
